# Activity-Dependent Synaptic Plasticity in *Drosophila melanogaster*

**DOI:** 10.3389/fphys.2020.00161

**Published:** 2020-02-25

**Authors:** Yiming Bai, Takashi Suzuki

**Affiliations:** School of Life Sciences and Technology, Tokyo Institute of Technology, Yokohama, Japan

**Keywords:** synaptic plasticity, activity-dependent neuroplasticity, *Drosophila melanogaster*, neuroplasticity, nervous system

## Abstract

The *Drosophila* nervous system is a valuable model to examine the mechanisms of activity-dependent synaptic modification (plasticity) owing to its relatively simple organization and the availability of powerful genetic tools. The larval neuromuscular junction (NMJ) in particular is an accessible model for the study of synaptic development and plasticity. In addition to the NMJ, huge strides have also been made on understanding activity-dependent synaptic plasticity in the *Drosophila* olfactory and visual systems. In this review, we focus mainly on the underlying processes of activity-dependent synaptic plasticity at both pre-synaptic and post-synaptic terminals, and summarize current knowledge on activity-dependent synaptic plasticity in different parts of the *Drosophila melanogaster* nervous system (larval NMJ, olfactory system, larval visual system, and adult visual system). We also examine links between synaptic development and activity-dependent synaptic plasticity, and the relationships between morphological and physiological plasticity. We provide a point of view from which we discern that the underlying mechanism of activity-dependent plasticity may be common throughout the nervous systems in *Drosophila melanogaster*.

## Introduction

Activity-dependent synaptic plasticity is a crucial component of activity-dependent neuroplasticity. Modern research has demonstrated that most of the neuroplasticity that occurs in our daily lives is synaptic. Synaptic plasticity is believed to be the most important neurological mechanism for learning and memory (Neves et al., 2008). Synaptic plasticity refers to the ability of synapses to increase or decrease their potential activity in response to environmental stimulation. It involves calcium influx (Zucker, 1999; Zhao et al., 2011), cell–cell communication (Dalva et al., 2007; Carrillo et al., 2010; Kochlamazashvili et al., 2010), reorganization of synaptic components (Packard et al., 2002; Packard et al., 2003; Okamoto et al., 2004; Sugie et al., 2015), localization of receptors (Raymond et al., 1993; Packard et al., 2002, 2003; Song and Huganir, 2002), regulation of pre-synaptic neurotransmission (Ho et al., 2011), autophagy (Liang, 2019), and many other biological processes. In brief, activity-dependent synaptic plasticity modulates how pre-synaptic neurons respond to activity-invoked physiological changes, and how post-synaptic neurons respond to changed neurotransmission from pre-synaptic neurons.

*Drosophila* is a powerful model organism that has been used to decipher numerous biological mechanisms over the past century (Stephenson and Metcalfe, 2013). Indeed, many important findings in higher organisms such as rats, mice, and humans are based on discoveries in *Drosophila*. The abundance of mutant lines and potent genetic tools, easy and rapid breeding, the applicability of these small organisms to imaging technologies and large-scale behavioral analysis, and conserved neurobiological mechanisms render *Drosophila* an ideal model for neuroscience research, including the study of synaptic function at the molecular, functional, and behavioral levels. Numerous studies have been performed on synaptic plasticity at the *Drosophila* neuromuscular junction (NMJ), and many molecules, proteins, and pathways implicated in NMJ plasticity have been subsequently shown to mediate similar functions at other synapses in *Drosophila* as well as in higher organisms. Findings on synaptic development at the *Drosophila* NMJ in the 1990s have inspired subsequent studies on synaptic refinement/plasticity in other systems, such as on the mechanisms involved in olfactory habituation and learning and memory. Some crucial pathways discovered in studies of synaptic development have also been found in the adult nervous system, which implies that similar mechanisms underlie synaptic development and plasticity.

Synaptic plasticity can be classified into two general types: (i) morphological (structural) plasticity involving branching, volume change, bouton formation, and alteration of synaptic contacts, and (ii) physiological (functional) plasticity involving regulation of neurotransmission, reorganization of synaptic components and receptors, and other processes regulating the strength of information flow between synapses. While a multitude of studies have suggested that morphological and physiological plasticity share many common mechanisms (Das et al., 2011; Yuan et al., 2011), some recent studies indicate that morphological and physiological plasticity may not be dependent on each other in the olfactory system, which renders the relationship complicated (Kidd and Lieber, 2016).

In this review, we describe activity-dependent synaptic plasticity in different parts of the *Drosophila* nervous system (larval NMJ, olfactory system, larval visual system, and adult visual system). We also discuss issues regarding the link between synaptic development and activity-dependent synaptic plasticity, the relationship between morphological and physiological plasticity, and potential common mechanisms underlying activity-dependent plasticity throughout the *Drosophila* nervous system.

## Pre-Synaptic Plasticity at the Larval Neuromuscular Junction

The relatively simple structure of the *Drosophila* nervous system and the availability of powerful genetic tools have facilitated the elucidation of various molecular components ubiquitously involved in synaptic modification, such as transcription factors, receptors, kinases and various effectors, and neuromodulators. The larval NMJ has been well-studied since 1990, and shown to be a highly representative model of synaptic development and plasticity. In the *Drosophila* NMJ, the sizes of pre-synaptic boutons, the number of active zones (AZs) in each bouton, and the complexity of the subsynaptic reticulum, a post-synaptic structure, increase during development (Lahey et al., 1994; Budnik, 1996). Refinement of neuronal connections during development, such as branching and regulation of synaptic components and receptors, resembles the process of neuroplasticity during critical periods after eclosion in adult flies (Golovin et al., 2019). Synaptic development is thus suggested to share signaling pathways and other mechanisms with neuronal plasticity, and discoveries made in studies of activity-dependent synaptic refinement during NMJ development have provided the foundation for subsequent studies on synaptic remodeling in various regions of the *Drosophila* nervous system (Sears and Broadie, 2017). Among those findings, the functions of the cAMP pathway and Wnt signaling pathway are particularly important.

### The Role of cAMP Pathway in Activity-Dependent Plasticity at the NMJ

*Drosophila* is a convenient model to explore the functions of genes, proteins, and biological processes because there are abundant mutant lines for nearly all genes (Jenett et al., 2012; Yamamoto et al., 2014). For elucidation of neuronal plasticity in *Drosophila*, it is common to study existing mutants or isolate new mutants with altered brain development or function. Keshishian and colleagues found that loss of electrical activity in pre-synaptic motor neurons in mutants with disrupted Na^+^ channel activity and in wild types treated with various toxins to prevent synaptic activity altered NMJ connectivity, which demonstrated that the development plasticity observed at the NMJ is activity-dependent. Loss of pre-synaptic activity increased inappropriate innervation of motor neurons onto muscle fibers (Jarecki and Keshishian, 1995). Alternatively, mutants in which neurons are hyperexcitable, such as *Shaker* (*Sh*) and *ether a go-go* (*eag*) (Burg and Wu, 1989), were examined at the NMJ, and it was found that during the pre-synaptic apparatus expansion stage, increased activity caused increased neurotransmitter release (Budnik et al., 1990).

**FIGURE 1:**
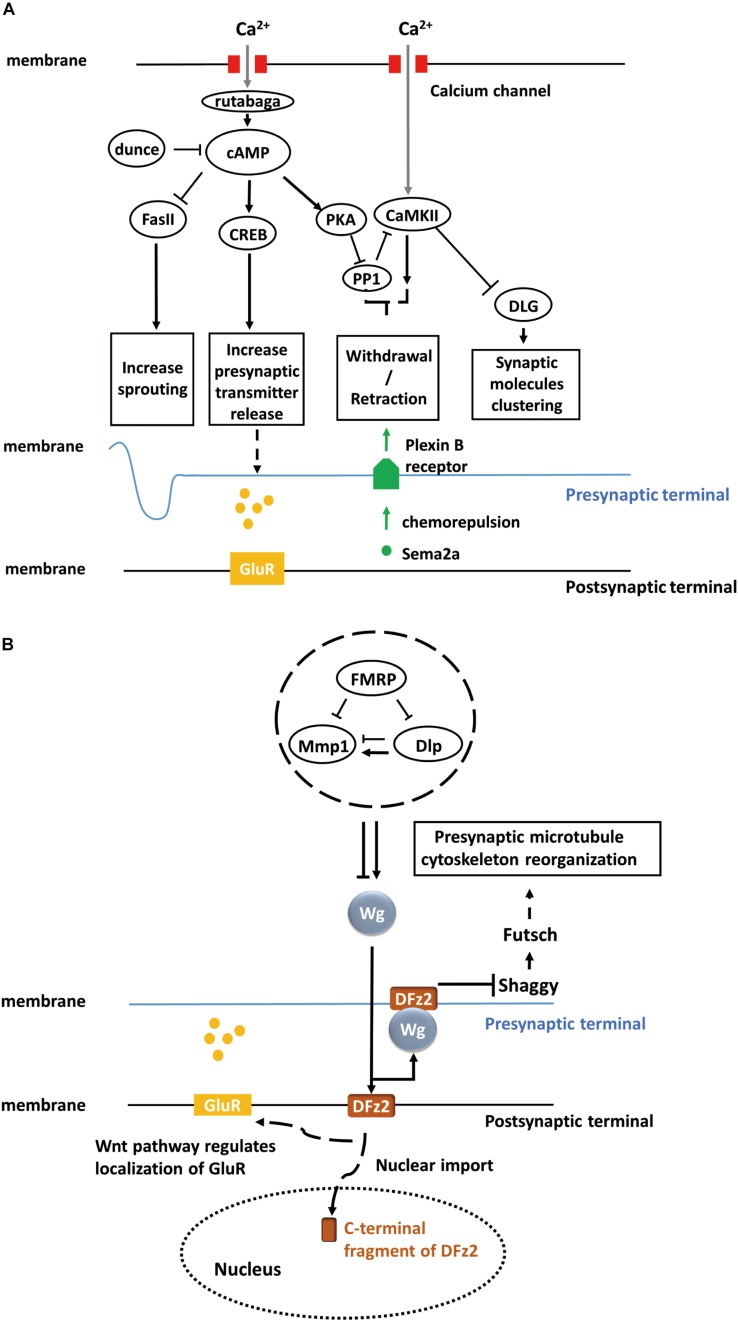
Activity-dependent synaptic plasticity at the *Drosophila* larval NMJ. **(A)** Calcium accumulation, along with neuronal activity, regulates the calcium/calmodulin-dependent adenylate cyclase, rutabaga. Dunce and rutabaga regulate the level of cAMP. Cell adhesion molecule Fas II and cAMP response element-binding proteins (CREB) are downstream of the cAMP pathway and responsible for increasing sprouting and pre-synaptic transmitter release. PKA and PP1 are also regulated by increased cAMP levels, and they, in turn, regulate CaMKII. The PKA-PP1-CaMKII interaction controls the withdrawal/retraction process through Plexin B receptor and Semaphorin-2a (Sema2a) chemorepulsion. The synaptic localization of Discs large (DLG) regulated by CaMKII controls the clustering of synaptic molecules, such as Fas II or Shaker. **(B)** The secretion of Wg proteins is enhanced in a calcium-dependent manner. The activation of a bidirectional Wg signaling pathway causes the nuclear import of the C-terminal fragment of Dfrizzled-2 (DFz2, receptor of Wg) at the post-synaptic terminal of NMJ and the rearrangement of pre-synaptic terminal structures involving the Shaggy/GSK-3β kinase, which controls the organization of the cytoskeleton and number of boutons through Futsch at the pre-synaptic terminal of NMJ. Moreover, activated Wnt pathway at the post-synaptic terminal also regulates the localization of glutamate receptors (GluRs) around synaptic boutons.

Fasciclin II (Fas II), a major cell adhesion molecule in pre-synaptic and post-synaptic membranes at the NMJ (Lin and Goodman, 1994; Zito et al., 1997), is necessary for the stabilization and growth of synapses and has an essential function in long-term synaptic structural plasticity, especially in the pre-synaptic apparatus (Schuster et al., 1996). The pre-synaptic sprouting phenotype of *Fas II* mutants resembles that of *eag Shaker* double mutants and *dunce* (cAMP phosphodiesterase II) mutants (Byers et al., 1981). *eag Shaker* and *dunce* increase neuronal activity and cyclic AMP (cAMP) concentration, respectively, and Fas II functions downstream (Schuster et al., 1996). However, *Fas II* mutation alone does not affect synaptic function and strength. Rather, the cAMP response element-binding protein (CREB) works cooperatively with Fas II to increase synaptic strength. Both the activation of CREB and downregulation of Fas II are cAMP-dependent and lead to increased pre-synaptic transmitter release (Figure 1A). cAMP pathway activation is induced by pre-synaptic calcium accumulation, which activates the calcium/calmodulin-dependent adenylate cyclase rutabaga (Livingstone et al., 1984; Levin et al., 1992).

Calcium/calmodulin-dependent protein kinase II (CaMKII) also contributes to activity-dependent plasticity through a separate pathway (Davis et al., 1996; Carrillo et al., 2010). Clustering of Discs large (DLG), which controls synaptic molecules such as Fas II, is regulated by CaMKII (Lahey et al., 1994; Zito et al., 1997; Koh et al., 1999) (Figure 1A). Moreover, rutabaga, cAMP-dependent protein kinase (PKA), and protein phosphatase 1 (PP1) act collaboratively with CaMKII to regulate chemorepulsion mediated by the muscle-secreted trans-synaptic chemorepellant Semaphorin-2a (Sema2a) during activity-dependent synaptic refinement, a process that serves to reduce aberrant neuromuscular connections (Figure 1A) (Vonhoff and Keshishian, 2017a,b).

To summarize, activity-dependent plasticity at the NMJ pre-synaptic structure of the NMJ requires mainly a calcium accumulation-induced, cAMP-dependent, second-messenger pathway, which involves crucial components, such as calcium/calmodulin-dependent adenylate cyclases (rutabaga), cell adhesion molecules (Fas II), and cAMP response element-binding proteins (CREB) (Figure 1A).

### The Role of Wingless in Activity-Dependent Plasticity at the NMJ

Wingless (Wg), a member of the Wnt family, is an important secreted protein involved in the development of *Drosophila* embryos. Wg is not only responsible for body segmentation (Pfeiffer et al., 2000) but also for the appropriate formation and arrangement of synapses. NMJ studies have shown that Wg is first secreted from pre-synaptic motor neuron terminals and endocytosed by post-synaptic muscles, where it triggers the Wnt signaling pathway in post-synaptic cells. The mutant *shibire* (*shi*) with deficient post-synaptic endocytosis exhibited post-synaptic Wg accumulation (Packard et al., 2002). Since Wg is necessary for embryonic development, late-stage larval mutants cannot be analyzed in these mutants. Therefore, a temperature-sensitive *wg* mutant was used to suppress Wg functions during the third instar stage. It was found that *wg* deficiency disrupted the distribution and localization of receptors, such as glutamate receptors (GluR), on the surface of post-synaptic terminals (Figure 1B). Further, *wg* deficiency can induce loss of both pre-synaptic and post-synaptic components. In summary, these studies indicate that Wg is an essential anterograde signal for the proper maturation of synapses at the NMJ.

Over the past decade, several studies have demonstrated that the bidirectional Wnt/Wg signaling pathway participates in activity-dependent synaptic structural and functional plasticity at the NMJ. Not only chronic but also acute activity alterations, along with development, shape the synapse structures (Packard et al., 2003). Rapid changes in synaptic structure dependent on acute activity alterations require the Wg signaling pathway. For example, the actin regulator Cortactin, present in both pre-synaptic and post-synaptic terminals of the NMJ, is a pre-synaptic regulator of rapid activity-dependent plasticity. Cortactin levels in stimulated pre-synaptic terminals which is necessary for activity-dependent plasticity requires the Wg pathway (Alicea et al., 2017). Acute activity of pre-synaptic neurons can enhance calcium-dependent Wg secretion and ensuing Wg signaling activity leads to (i) nuclear import of the C-terminal fragment of the Wg receptor Dfrizzled-2 (DFz2) in the post-synaptic terminals of NMJs and (ii) rearrangement of pre-synaptic terminal structures involving the Shaggy/GSK-3β kinase, which controls the organization of the cytoskeleton and the number of boutons at NMJs (Ataman et al., 2008) (Figure 1B). In the illustrated study, GFP was used to tag pre-synaptic and post-synaptic components such as GluRs (Heckscher et al., 2007) and Bruchpilot (Brp) (Wagh et al., 2006), and live-imaging technology was utilized to examine dynamic changes in these components under changes in activity. Moreover, Wg was shown to inhibit Shaggy and regulate cytoskeletal reorganization through the microtubule-associated protein Futsch (Miech et al., 2008). In conclusion, Wg, Shaggy, and Futsch form Wnt/Wg signaling pathway and regulate the cytoskeleton reorganization (Figure 1B).

Fragile X mental retardation protein (FMRP), an RNA-binding translational repressor (Darnell et al., 2001), has recently been demonstrated to regulate the Wnt/Wg signaling pathway. Extracellular matrix metalloproteinase (MMP) and the heparan sulfate proteoglycan (HSPG) Dally-like protein (Dlp), a Wg co-receptor (Khare and Baumgartner, 2000; Kirkpatrick et al., 2004), work together to regulate rapid activity-dependent synaptic bouton formation (Dear et al., 2017). Dlp can upregulate or downregulate the Wg pathway depending on the relative abundance of Wg pathway components. FMRP-MMP-Dlp play an essential role in activity-dependent synaptogenesis via Wnt/Wg trans-synaptic signaling pathway (Dear et al., 2017; Sears and Broadie, 2017) (Figure 1B).

To summarize, Wingless is responsible not only for synaptic maturation but also for activity-dependent synaptic structural and functional plasticity at the NMJ. Wnt/Wg signaling pathway composed of Wingless, Shaggy, and Futsch regulates the rearrangement of pre-synaptic terminal structures in an activity-dependent manner. The distribution and localization of receptors in the post-synaptic terminals are also regulated by Wg. FMRP, MMP, and Dlp cooperate to modulate the level of Wg and act as upstream regulators of Wnt/Wg signaling.

## Synaptic Plasticity in the Olfactory System

The *Drosophila* olfactory system has long been regarded as an accessible model for studying the development and plasticity of a primary sensory system owing to its well-described anatomical structure. The olfactory system is particularly attractive for studies on structural and functional plasticity related to behavior as it is the locus of a reliable odorant habituation behavior. Olfactory sensory neurons (OSNs) distributed in the antenna and maxillary palps receive odor information through odorant receptors (Miazzi et al., 2016) and then project axons to glomeruli in the antennal lobe (AL), where they form synapses with projection neurons (PNs) or local interneurons (LNs). The PNs of the inner antennocerebral tract (iACT) send axons to the mushroom body (MB) synapse with Kenyon cells in the calyx, and finally terminate in the lateral horn (LH). Alternatively, PNs of the medial antennocerebral tract (mACT) project directly to the LH from glomeruli (Keene and Waddell, 2007; Golovin and Broadie, 2016).

Numerous studies have been conducted on activity-dependent AL plasticity during critical periods after eclosion, including studies on glomerulus volume changes (Sachse et al., 2007) and short-term or long-term habituation (Das et al., 2011). Findings from activity-dependent plasticity studies in AL have benefited from previous studies on other *Drosophila* nervous systems, particularly the NMJ, since activity-dependent AL plasticity involves many similar signaling pathways. However, studies of the olfactory system have also revealed novel plasticity mechanisms. Activity-dependent AL plasticity involves the cAMP pathway (Das et al., 2011), translational regulation (McCann et al., 2011; Sudhakaran et al., 2014), central *N*-Methyl-D-aspartate (NMDA) glutamatergic signaling pathway (Das et al., 2011), and the Notch pathway (Kidd and Lieber, 2016). While the Wnt signaling pathway does not appear to be involved in AL plasticity during the critical period, Shaggy contributes to glomerulus remodeling through other pathways (Acebes et al., 2011; Golovin et al., 2019).

A major form of morphological plasticity in AL is the increased volume of the V glomerulus in the antennal lobe induced by early long-term exposure to CO_2_ after eclosion (Sachse et al., 2007). This increase is reversible after returning the flies to ambient conditions, indicating a highly flexible synaptoplastic mechanism. Based on experiments with *Drosophila* expressing the genetically encoded calcium indicator GCaMP in the V glomerulus, this plasticity was shown to be activity-dependent. Prolonged exposure to CO_2_ also increased the activity of LNs in the V glomerulus, especially inhibitory LNs, which in turn enhanced the inhibitory effects of gamma-aminobutyric acid (GABA) released from LNs onto PNs (Wilson and Laurent, 2005; Liu and Wilson, 2013). As a result, the output of PNs to the LH should be reduced, a notion validated by Sachse et al. (2007) in a study expressing GCaMP in PNs from the V glomeruli. These authors also found that there is a critical period after eclosion for this olfactory plasticity (Sachse et al., 2007; Golovin et al., 2019).

It was also shown that long-term exposure to CO_2_ can selectively reduce the subsequent behavioral responses to CO_2_, termed habituation (Sachse et al., 2007). Olfactory habituation, including short-term habituation (STH) and long-term habituation (LTH), requires rutabaga-encoded adenylate cyclase, which is induced by calcium accumulation and G protein-coupled receptor (GPCR) activation (Das et al., 2011). The upregulation of rutabaga in LTH induces cAMP signaling in inhibitory GABAergic LNs, resulting in reduced PN activity. Moreover, NMDA receptors in post-synaptic PNs (Schoppa et al., 1998) are responsible for the odorant selectivity of olfactory habituation. Both STH and LTH were blocked when GABA and glutamate release from LN1 neurons was impeded through RNAi-based knockdown of the GABA synthesis enzyme glutamic acid decarboxylase (GAD1) (Ng et al., 2002) and the vesicular glutamate transporter DVGLUT (Daniels et al., 2008). Thus, co-release of GABA and glutamate from LN1 neurons is essential for olfactory habituation.

Activation of the cAMP downstream transcription factor CREB2 in LNs is required for LTH, but not for STH, indicating that LTH requires additional components compared with STH. Ataxin-2 (Atx2), an RNA regulation-related protein, works with miRNA components such as Me31B and Argonaute 1 (Ago1) in PNs to regulate olfactory LTH (McCann et al., 2011). The Atx2-involved miRNA pathway represses mRNA translation via the Ago1-RNA-induced silencing complex (Ago1-RISC). Interestingly, Ago1-RISC in olfactory PNs requires FMRP, which also contributes to Wnt/Wg signaling during activity-dependent NMJ plasticity. FMRP, together with Atx2 and Ago1, functions to repress CaMKII expression (Sudhakaran et al., 2014) in both pre-synaptic inhibitory LNs and post-synaptic PNs, and this suppression is required for olfactory LTH, although the underlying mechanism remains obscure. Recent studies show that CaMKII is responsible for spontaneous release, which implies that reduced spontaneous transmitter release contributes to LTH maintenance (Kuklin et al., 2017).

While morphological structural plasticity and physiological habituation in the olfactory system occur almost simultaneously and share some fundamental underlying mechanisms such as dependence on rutabaga, DVGLUT, and NMDA receptors, they still have differences. For instance, GABA_A_ receptors appear unnecessary for the volume change in the glomerulus after long-term exposure to odors. Instead, GABA_A_ receptors may be necessary for physiological rather than structural plasticity (Das et al., 2011). Moreover, the non-canonical Notch signaling pathway is implicated in glomerulus structural plasticity (volume changes) while physiological plasticity requires only the canonical Notch signaling pathway (Kidd and Lieber, 2016).

## Post-Synaptic Plasticity in Larval Visual System Branching

In the *Drosophila* larval visual system, Bolwig’s organ (BO) functions as the light sensing organ. BO sends information to ventral lateral neurons or LN(v)s through Bolwig’s nerve (BN), which terminates in a region rich in LN(v) dendrites (Malpel et al., 2002; Farca-Luna and Sprecher, 2013). Using the green fluorescent protein reconstitution across synaptic partners (GRASP) technique, it was shown that LN(v)s are post-synaptic to BN (Feinberg et al., 2008; Yuan et al., 2011). Upon light stimulation, LN(v)s are activated by BN, and BO maintains LN(v) dendrites. Light exposure hinders the growth or branching of LN(v) dendrites during larval visual system development, and this plasticity requires pre-synaptic BO input instead of post-synaptic LN(v) light sensing function. Excitation of the BO or LN(v)s can decrease the dendrite length of LN(v)s. In addition, expression of certain pre-synaptic terminal components, such as the calcium channel Cacophony (Kawasaki et al., 2004), are downregulated in the presence of light stimulation, resulting in loss of synaptic connection between BN and LN(v)s.

The cAMP phosphodiesterase II dunce also participates in the plasticity of post-synaptic LN(v) branching. In the *Drosophila dunce* mutant, larval LN(v)s do not exhibit significant differences in length under changing light conditions. Further, experiments in which post-synaptic LN(v)s express the catalytic subunit of protein kinase A (PKAmc) or a dominant-negative form of CREB (CREBdn) to up- or down-regulate cAMP levels demonstrated that cAMP levels are essential for modifying the structure and function of LN(v)s (Yuan et al., 2011). Moreover, babos-1, a cell surface protein containing the extracellular immunoglobulin domain, also participates in regulation of plasticity, but it remains unknown how it controls dendrite length.

To summarize, structural and functional plasticity at post-synaptic LN(v)s requires both LN(v) activity and light-induced pre-synaptic BN activity. The cAMP signaling pathway and cell surface proteins such as babos-1 in post-synaptic LN(v)s also play essential roles in this modification for light adaption.

## Pre-Synaptic Plasticity in the *Drosophila* Adult Visual System

The visual system of *Drosophila* is composed of the retina and optic lobe. The optic lobe is composed of the lamina, medulla, lobula, and lobula plate. There are around 750 small eyes called ommatidia in the retina, and each ommatidium has eight photoreceptor neurons (R1 to R8). Photoreceptor neurons R1-R6 innervate the lamina layer, whereas R7 and R8 innervate the medulla layer. Photoreceptor neurons mainly release histamine to post-synaptic neurons. Previous studies showed that activity-dependent remodeling of central synapses occurs with natural stimuli, and this also applies to the *Drosophila* adult visual system. AZ components in photoreceptor neurons were found to be reorganized depending on activity, and the Wnt pathway is involved in this process.

### Activity-Dependent Reorganization of AZ Components in Photoreceptor Neurons

It was found that some AZ components, including Bruchpilot (Brp), DLiprin-α, and the conserved RIM-binding protein DRBP, which are crucial for arranging synaptic vesicles and calcium channels, can be redistributed after long-term exposure to light in R8 photoreceptor neurons (Sugie et al., 2015). Endogenous Brp in R8 photoreceptor neurons was labeled via synaptic tagging by the recombination (STaR) method (Chen et al., 2014). When flies were kept in constant light (LL) for 1–3 days after eclosion, the expression of Brp in each R8 photoreceptor was significantly reduced compared to flies kept under normal 12-12-h light-dark (LD) conditions. The same result was observed when fluorescently tagged Brp-short-mcherry was used to label Brp. Electron microscopy (EM) revealed that the number of T-bars in the AZ also decreased after LL compared to LD and as a result eliminated transmitter release from R8. The authors also tagged other AZ components, such as DLiprin-α, DRBP, Dsyd-1, and Cacophony (Cac), using GFP and found that DLiprin-α and DRBP were reorganized after LL, whereas Dsyd-1 and Cac remained unaffected.

To examine whether the changes in AZ are activity-dependent, they used temperature-sensitive shibire [UAS-shi(ts)] (Kitamoto, 2001) to restrict the activity of R8 photoreceptor neurons and found that the reorganization of Brp after LL was suppressed. Moreover, post-synaptic histamine receptor mutants also suppressed the loss of Brp after LL. These results indicate that both pre-synaptic photoreceptor and post-synaptic second-order neuron activity via histamine receptor modulate Brp localization.

### Pathways Involved in Activity-Dependent Reorganization of AZ Components in Photoreceptor Neurons

The divergent canonical Wnt pathway is involved in this activity-dependent reorganization of AZ components after continuous light exposure. Canonical Wnt pathway-related proteins, such as Arrow (Arr), a Wnt co-receptor with Frizzled-2 (He et al., 2004), Dsh, a cytosolic phosphoprotein (Clevers, 2006), and Shaggy all contribute to the maintenance AZ component localization. Since Shaggy phosphorylates Fustch, which is involved in microtubule stabilization, and Shaggy is negatively regulated by the Wnt pathway (Miech et al., 2008), Wnt signaling may regulate reorganization of AZ components through microtubule destabilization. Indeed, further experiments on AZ component localization after directly disturbing microtubule stabilization revealed that the delocalization of AZ components can be ascribed to microtubule destabilization (Sugie et al., 2015).

When faced with environmental changes or stressors, cells will activate the unfolded protein response (UPR) (Ron and Walter, 2007) in the ER as a protective mechanism, a process that involves adenylylation of the core UPR regulator BiP (Bertolotti et al., 2000). After flies were treated with constant light for 72 h, photoreceptors with mutations in UPR-related proteins, such as BiP, severely lost synaptic function and this loss was reversible once these mutant flies were returned to a normal 12-12-h LD cycle (Moehlman et al., 2018). This finding demonstrates that cellular homeostasis and adenylylation may be involved in the activity-dependent plasticity of photoreceptor pre-synaptic functions.

To summarize, continuous light exposure in adult *Drosophila* leads to activation of pre-synaptic photoreceptors and hyperpolarization of post-synaptic neurons. The Wnt/Wg pathway in photoreceptors induces microtubule destabilization, which causes the delocalization of AZ components and finally the loss of synaptic connections between photoreceptors and second-order neurons. Homeostatic protective mechanisms also act to prevent degeneration of photoreceptors during continuous light exposure.

## Conclusion and Future Directions

Huge strides have been made on understanding activity-dependent synaptic plasticity at the *Drosophila* NMJ and in the olfactory system. This progress has benefited from the well-studied anatomic structure of the *Drosophila* nervous system. The anatomical information provides details about neuronal connections that have allowed researchers to identify where connections are changed or lost under specific conditions or in organisms with specific mutations. Even though the *Drosophila* adult visual system has well-described anatomical structures and connectome, the mechanisms underlying activity-dependent plasticity are still largely unclear. Nonetheless, the *Drosophila* adult visual system warrants further study since it possesses complex layered structures that resemble those of mammals.

Early studies on synaptic maturation at the NMJ have laid the cornerstone for subsequent research on other regions of the *Drosophila* nervous system. Molecular and functional pathways described at NMJ, such as cAMP and Wingless, participate in many activity-dependent synaptoplastic process in other regions, such as *Drosophila* sensory systems. The similarity between the development of neuronal connections and synaptic plasticity suggests that synaptic plasticity may be a recapitulation of synaptic development, so knowledge gained on synaptic development should guide studies on activity-dependent plasticity.

Studies on the olfactory system show that while activity-dependent morphological and physiological plasticity share some components such as rutabaga, NMDA receptors and DVGLUT in olfactory system, there may be no direct connection between them since they require different Notch pathways. Two types of plasticity, morphological and physiological, or structural and functional, often occur simultaneously, but in some cases these events appear independent. Thus, caution is advised when extrapolating conclusions drawn from studies on morphological plasticity to physiological plasticity and vice versa.

**FIGURE 2:**
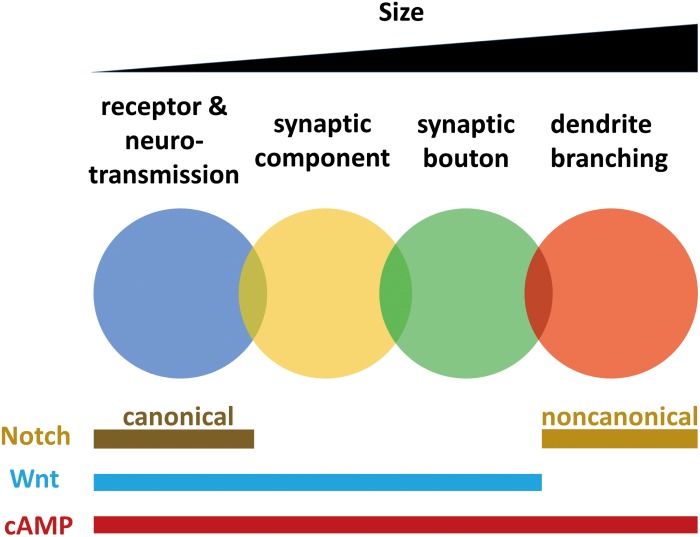
Mechanisms of synaptic plasticity working at different scales from neurotransmission to branching are overlapped and interwoven. Synaptic plasticity occurs in different scales, including receptors and neurotransmission, synaptic components, synaptic boutons and dendrites. Localization of receptors and regulation of neurotransmission, synaptic component reorganization, bouton formation and dendrite branching all require cAMP pathway. Dendrite branching does not need Wnt pathway, while plasticity at other smaller scales require Wingless. Neurotransmission requires canonical Notch pathway, while dendrite branching requires non-canonical Notch pathway.

Localization of receptors and regulation of neurotransmission, synaptic component reorganization, bouton formation, and dendrite branching all require the cAMP pathway. Dendrite branching does not require the Wnt pathway (Golovin et al., 2019), but synaptic plasticity at other smaller scales requires Wingless. Neurotransmission and dendrite branching require different Notch pathways. These findings indicate that mechanisms of synaptic plasticity working at different scales from neurotransmission to branching are not isolated but are overlapped and interwoven (Figure 2).

Nonetheless, there are mechanisms common to activity-dependent plasticity among regions. Crucial pathways at the NMJ include (i) the calcium accumulation-induced cAMP-dependent second-messenger signaling pathway, which involves rutabaga and CREB, (ii) cell adhesion molecules such as Fas II, and (iii) the Wnt/Wg signaling pathway. Olfactory habituation (Sato et al., 2018) and post-synaptic plasticity in the larval visual system also involve the cAMP pathway. The Wnt signaling pathway also functions in adult visual system plasticity by regulating microtubule stabilization. FMRP is crucial for modulating the Wnt pathway at the NMJ and also cooperates with Ago1-RISC to suppress CaMKII expression for long-term olfactory habituation. NMJ plasticity, olfactory habituation, post-synaptic plasticity in the larval visual system, and pre-synaptic plasticity in the adult visual system all require reorganization of synaptic components and regulation of neurotransmitter release.

**FIGURE 3:**
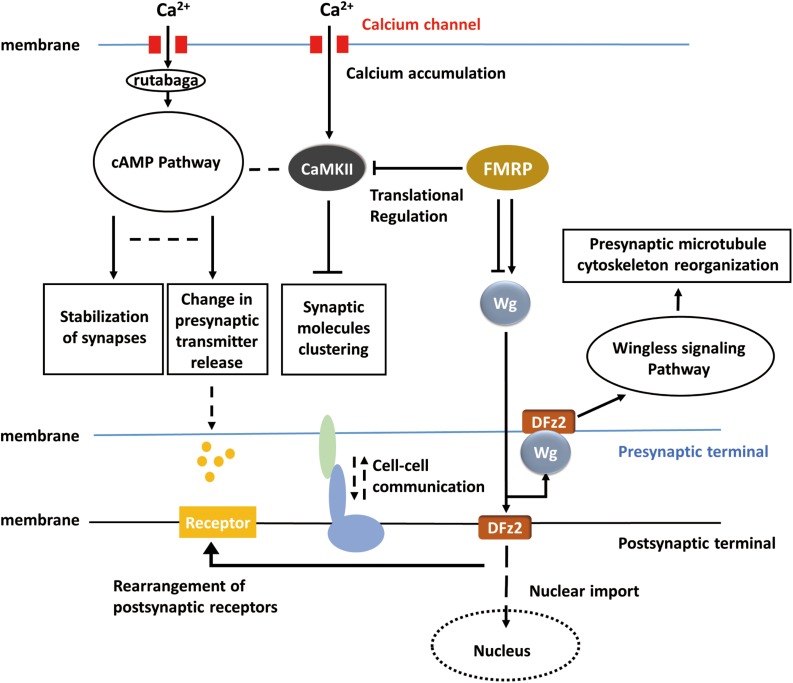
Hypothetical universal model for activity-dependent plasticity. Calcium accumulation invokes the cAMP pathway and CaMKII pathway. Translational regulation (RNA-induced silencing complex, RISC) modulates CaMKII and the Wnt/Wg pathway. Wg pathway is responsible for pre-synaptic microtubule destabilization and rearrangement of post-synaptic receptors, whereas cAMP pathway is responsible for the change in neurotransmitter release. CaMKII, cAMP, and Wg pathway reorganized pre-synaptic components together. The bidirectional regulation of synaptic plasticity also requires cell–cell talk/communication.

It is intriguing that many of the underlying mechanisms for activity-dependent plasticity may be common throughout the nervous systems in *Drosophila*. A big picture of activity-dependent synaptic plasticity can be drawn, and the apparently lost pieces according to the general view can in return provide some promising directions for the study in activity-dependent synaptic plasticity. For instance, in the adult visual system, the cAMP pathway, RISC, cell adhesion molecules, and similar cell surface proteins may also be required but related studies are absent so far. In the big picture (Figure 3), when activity occurs, calcium influx activates cAMP pathway and in result reorganizes pre-synaptic components and regulates the release of neurotransmitters. Calcium accumulation also affects clustering of synaptic molecules via CaMKII. FMRP regulates the translation of CaMKII and involves in Wingless signaling pathway which controls reorganization of pre-synaptic microtubule cytoskeleton. However, the molecules upstream of the Wnt or FMRP pathways remain unidentified, and the link between neuronal activity and Wg signaling requires further studies. At the post-synaptic terminal, neurotransmitter receptors can be correspondingly rearranged according to pre-synaptic activity, and this process may be dependent on Wingless signaling pathway. It remains unclear whether cAMP pathway is also involved in the rearrangement of post-synaptic receptors or not. Furthermore, will the activity of post-synaptic sides affect the synaptic component organization or neurotransmitter releasing in the pre-synaptic sides? Some sorts of cell–cell communications may exist between pre-synaptic and post-synaptic terminals, which possibly coordinate the morphological and physiological changes on both sides, but related studies are missing in *Drosophila*. It is believed that activity-dependent synaptic plasticity requires the participation of both pre-synaptic and post-synaptic sides. However, the mechanisms of ‘feedback’ from post-synaptic side are not well-studied in *Drosophila*. Conclusions, techniques, and experiences from previous studies may inspire the exploration of activity-dependent synaptic plasticity in *Drosophila melanogaster* and complete the entire picture.

## Author Contributions

YB designed, wrote, and revised the manuscript and prepared the figures. TS designed, revised, and approved the manuscript and figures.

## Conflict of Interest

The authors declare that the research was conducted in the absence of any commercial or financial relationships that could be construed as a potential conflict of interest.
